# Validation of Training and Acquisition of Surgical Skills in Veterinary Laparoscopic Surgery: A Review

**DOI:** 10.3389/fvets.2020.00306

**Published:** 2020-06-03

**Authors:** Carlos A. Oviedo-Peñata, Angelo E. Tapia-Araya, Juan D. Lemos, Carlos Riaño-Benavides, J. Brad Case, Juan G. Maldonado-Estrada

**Affiliations:** ^1^Tropical Animal Production Research Group, Faculty of Veterinary Medicine and Zootechny, University of Cordoba, Monteria, Colombia; ^2^Surgery and Theriogenology Branch OHVRI-Group, College of Veterinary Medicine, University of Antioquia, Medellin, Colombia; ^3^LaparoEndoVet, Mobile Laparoscopy and Endoscopy Service, Barcelona, Spain; ^4^Bioinstrumentation and Clinical Engineering Research Group (GIBIC), Bioengineering Department, Engineering Faculty, Universidad de Antioquia, Medellín, Colombia; ^5^Department of Small Animal Clinical Sciences, College of Veterinary Medicine, University of Florida, Gainesville, FL, United States

**Keywords:** laparoscopy, minimally invasive surgical procedures, simulation training, veterinary surgery, veterinary education

## Abstract

At present, veterinary laparoscopic surgery training is lacking in experiences that provide a controlled and safe environment where surgeons can practice specific techniques while receiving experts' feedback. Surgical skills acquired using simulators must be certified and transferable to the operating room. Most models for practicing laparoscopic skills in veterinary minimally invasive surgery are general task trainers and consist of boxes (simulators) designed for training human surgery. These simulators exhibit several limitations, including anatomic species and procedural differences, as well as general psychomotor training rather than *in vivo* skill recreation. In this paper, we review the existing methods of training, evaluation, and validation of technical skills in veterinary laparoscopic surgery. Content includes global and specific scales, and the conditions a structured curriculum should meet for improving the performance of novice surgeons during and after training. A focus on trainee-specific assessment and tailored-technical instruction should influence training programs. We provide a comprehensive analysis of current theories and concepts related to the evaluation and validation of simulators for training laparoscopic surgery in small animal surgery. We also highlight the need to develop new training models and complementary evaluation scales for the validation of training and acquisition of basic and advanced skills in veterinary laparoscopic surgery.

## Introduction

Incorporation of routine minimally invasive surgery (MIS) training is a continuing matter in human (1–3) and veterinary surgery (4–6). The limited availability of models for routinely scheduled training in minimally invasive techniques in veterinary surgery is a fundamental problem to be solved for achieving the goal of well-trained veterinary MIS surgeons (7, 8). The application of MIS started at the same time in humans and small animals, but the latter appears to be delayed 20 years if compared to the humans' counterpart (9). Laparoscopy is one of the most commonly practiced MIS disciplines providing advantages in the recovery of patients in small animal surgery compared to conventional surgery (9–13). However, laparoscopic surgery has disadvantages such as the cost of equipment and a longer and more complex learning curve that must be developed gradually and ethically through the use of simulators and training models (10). These facts make it difficult for its massive implementation, particularly at the educational level.

Only recently has the importance of developing models of simulation for training and improving the skills of veterinary surgeons in laparoscopic surgery been recognized (8, 14–16). Many technical differences exist between conventional and laparoscopic surgery (8, 16–18), and these differences render the traditional model of teaching surgery inadequate for teaching and training laparoscopic surgery (19–21). The existing methods of simulation in surgery were created to reduce the learning curve in a controlled and safe environment (21). Therefore, it is considered a valuable educational method for teaching and training veterinary medicine students with basic and advanced surgical skills (14, 17). Deliberate practice is a regimen of activities designed to optimize the improvement of skills in this surgical case at an expert level, providing that essential aspects such as repetitions and feedback are met, with clear objectives to reach the thresholds set for each task (22). Although the time of acquisition, development, and improvement of cognitive and motor skills is subject to the innate psychomotor skills of each individual (23), the training strategies have been constructed in terms of training time, distribution of sessions and number of hours, according to each program and tasks assigned objectively in order to improve the quality of care and safety (24). For this, criteria of competence are defined that allow establishing the optimal level that is desired to be obtained by each student, in order to achieve competent surgeons capable of integrating the knowledge, skills and attitudes to carry out an activity during training (25).

Widely validated training programs such as the Fundamentals of Laparoscopic Surgery (FLS) have been used for the evaluation of training in laparoscopic surgery (9, 17, 20), and the laparoscopic simulation training program called Veterinary Assessment of Laparoscopic Skills (VALS) (25). These programs allow the development of both basic and advanced skills, and the exercises are organized sequentially on an ascending scale of difficulty (26). In the training plans, it is essential that the students receive the necessary instructions and corrections to avoid making mistakes during the training, and thus, guarantee their adequate understanding and performance (27). A critical point within the training process is the assessment of skills. This can be done using motion capture devices or through scales to know the progression of surgical skills and errors. Therefore, assessment instruments, simulators, and even the training plan must meet the validity and reliability criteria that support their use (21, 28).

Accordingly, this review was carried out starting from the question: Are there relevant and critical aspects for the construction of a training, evaluation, and validation plan in veterinary laparoscopic surgery? Interest was focused on manuscripts related to medical education in laparoscopic surgery in both human and veterinary medicine. The literature search was performed by searching in the PubMed electronic databases (https://www.ncbi.nlm.nih.gov/pubmed/), SciELO—Scientific Electronic Library Online (https://scielo.org/en), and Google Scholar (https://scholar.google.com/) dates from January 2019. Terms such as “laparoscopic skill in veterinary” and “teaching,” or “training surgical skills” and “simulation,” or “assessment or validity in laparoscopy” were searched.”

## Curriculum Notions of Training in Veterinary Laparoscopic Surgery

### Learning Theories in Medical Education and the Acquisition of Surgical Psychomotor Skills Applied to Surgical Simulation

Several theories have been proposed for supporting the conceptual framework of learning and training of surgical skills, particularly for simulation in MIS. The classical method for teaching surgical skills in human and veterinary medicine is the tutor-student model introduced by Halsted in 1889 for training novice surgeons in Johns Hopkins Hospital, which is based on the principle of learning under the supervision of an experienced surgeon (28).

There are multiple hypotheses about the acquisition of cognitive and psychomotor skills in medical simulation. However, one of the theories that underpin the use of simulation on adult learning is the Theory of Kolb (24), which is based on “one's own experience and reflection.” Learning begins with the student's experiences of an event that will reflect what happens later when it is finally conceptualized. At the right moment, students will experiment and realize their own experience. This sequence of actions is followed by the compression of the activity when it is established mentally and practiced repeatedly but making the appropriate corrective measures to improve it. Another hypothesis is the theory of change by Kurt Lewin, who explains the reasons why individuals seek educational experiences for improving their skills. The process happens in three stages: (1) Defrosting, which begins once an individual becomes aware of the error or misconduct, resulting in a feeling of discomfort that promotes the desire to change. (2) The change, which will allow individuals to adopt new knowledge, skills, or attitudes in their careers. And (3) Freezing, which occurs once new knowledge is adopted in mind (27). Russell and Barrett (29), developed the circumflex model that exerted a valuable impact in simulation, because the use of this didactic strategy implies a high degree of emotionality, especially in medium and high fidelity simulation. Accordingly, emotions are distributed in a circular two-dimensional space, which contains dimensions of excitement-deactivation and pleasure-displeasure. The scenarios of clinical simulation must achieve enough emotional stimulation to keep the student in a state where the elements corresponding to the quadrants of pleasure and activation predominate for achieving meaningful and lasting learning. In the theory-based learning model by Kneebone (30), active learning must be based on simulation, including actual clinical practice, and considering four essential aspects: (1) The gain and retention of technical competence, through sustained and deliberate practice in a safe environment, in order to consolidate recently acquired skills. (2) Accompanying by expert tutors in task-based learning, when appropriate, which progressively decreases as apprentices improve their skills. (3) Learning in a professional real-life context. And (4) The affective component provides a supportive, motivating, and student-centered environment conducive to learning.

For the development of psychomotor skills, one of the most recent training techniques is based on the Fitts and Posner's theory of the acquisition of motor skills (1967) (31), which comprises three stages of knowledge: Cognition, Integration, and Automation. In the cognition stage, the assigned activity is developed using the explanation and demonstration of a task, and the apprentice's objective is to intellectualize it; for this stage, the performance on basic skills is erratic, and the procedure is carried out in several steps. The integration stage is developed with deliberate practice and feedback, having as an objective to understand the specific task and perform it mechanically; that is, the knowledge is translated into appropriate motor behavior. At this stage, the learners are still thinking about how to move, but they can execute the task more fluently and with fewer interruptions. In the automation stage, the practical activity results in a refining and automated performance that requires little cognitive information (previously acquired). The goal is accomplished when the apprentice no longer needs to think about how to execute the assigned task, performing it with speed, efficiency, and precision (31–33). According to the theory of deliberate practice by Ericsson (1993), most professionals can reach a stable and average level of performance, which they maintain throughout their careers (22). The deliberate practice is considered a critical process for the development of a domain or experience. As an example, the number of hours devoted to deliberate practice, instead of just the hours dedicated to surgery, is a crucial determinant of the level of experience (31). Deliberated practice should be structured into four main criteria (32) to achieve significant and uniform improvements in individuals' performances. (1) Assignment to a task with a well-defined objective and identified by an instructor. (2) Motivation to improve, that must allow enough focus to maintain active efforts for improving the performance. (3) Providing feedback, in which the trainee must receive valid comments about their performance and suggestions focused on mistake correction. And (4) Providing opportunities for repetition and gradual refinements of performance within a controlled environment. The level of experience achieved by trainees is closely related to the time devoted to deliberate practice in expert athletes, chess players, and musician's performances (22, 31).

Considering that, improving the surgeon's skills with premeditated and autonomous training is mandatory for successful performance during laparoscopic surgery. Gallagher et al. (23) proposed a conceptual framework for facing the learning modules required for MIS training, which is based on the concept of finite individual capacity for attention (see Table 1). The attention capacity that allows the mental powers to focus on an object or task (listening, observing, concentrating), is limited in humans.

**Table 1:** Theories supporting the conceptual framework of learning and training of surgical skills.

**Theory/model**	**Author/Year**	**Principle**	**Goal**	**References**
Tutor-student model	William S. Halsted/1889	Training under the advisory of an experienced surgeon.	Find the most efficient way to learn psychomotor and cognitive skills.	(28)
Theory of Kolb	David Kolb/1974	Own experience-based learning supported on two levels: the four-stage cycle of learning and four different learning styles.	Adult learning in each one's own experience and reflection.	(24)
Lewin's theory of change	Kurt Lewins	Stages: (1) Defrosting when the individual becomes aware of the error or misconduct. (2) The change, allowing individuals to adopt new knowledge, skills, or attitudes. (3) Freezing, which occurs once new knowledge is realized in mind.	Individuals seek an educational experience to improve.	(27)
Circumflex model	Russel JA, Barret LF/1999	Emotions are distributed in a circular two-dimensional space, which contains dimensions of excitement-deactivation and pleasure-displeasure.	Achieve enough emotional stimulation to keep the student in a state where predominates the elements corresponding to the quadrants of pleasure and activation.	(29)
Theory-based learning	Kneebone /2005	(1) Gain and retention of technical competence. (2) Access to expert tutors when appropriate. (3) Learning within a real-life context. (4) The affective component provides a student-centered environment conducive to learning.	Effective learning based on simulation and engaged in real-life practice.	(30)
Theory of acquisition of motor skills	Fitts and Posner/1967	Cognition, Integration, and Automation.	The apprentice no longer needs to think about how to execute the assigned task.	(31)
Gradual improvement of performance	Ericsson/1993	(1) Undertake a well-defined objective task identified by an instructor. (2) Motivation to improve, maintaining active efforts for improving the performance. (3) Provided with feedback, the trainee must receive valid comments. (4) Provided with ample opportunities for repetition and gradual refinements.	Achieve significant and uniform improvements in the performance of individuals.	(22)
Concept of finite individual capacity for attention	Gallagher/2005	Expand the buffer zone of attentional resources.	The automation of fundamental psychomotor skills	(23)

**Figure 1:**
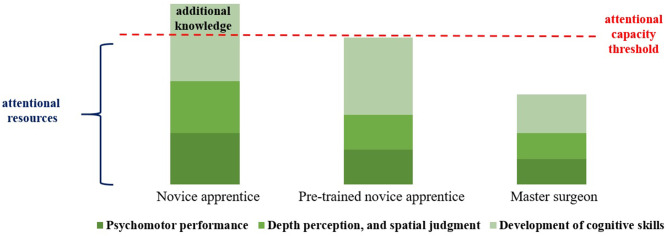
Benefits of hypothetical attentional resources of simulation training described by Gallagher et al. (23). Adapted from Choy and Okrainec (34).

For this reason, only a finite amount of information or stimuli can be attended to at any given time. The care resources used during a procedure vary according to surgical experience. Therefore, an experienced surgeon occupies less attention capacity for psychomotor activities, depth perception, spatial location, surgical judgment, and decision-making as opposed to a less experienced surgeon. This represents a larger buffer zone that is used to improve cognitive abilities (such as controlling complications during a surgical procedure). As trainees in laparoscopic surgery use these attention resources to control their hands during training, their complementary attention capacity is limited, and the attention threshold of the apprentices is quickly exceeded. Ultimately, the idea is to get “pre-trained newbies” who spent significantly less attention resources through simulation to develop automated technical skills in a non-surgical setting (34), so they will be able to focus more on learning the steps of the operation and learning how to manage complications once they enter the clinical setting (23) (see Figure 1).

### Methods of Verbal Correction During the Training Process

The simulated surgical training becomes a complement for the apprentices during their professional training process, before the clinical encounter creating a safe training environment to gain experience progressively with no or minimal risk for the real patient. It also shortens the learning curve, preventing the surgeon from completing this curve in the operating room, and allows the performance of training programs based on flexible competition, progressively according to the specific requirements of surgeons or institutions (35). There is an implicit assumption that simulators teach good behaviors (doing the task correctly) from the configuration of the training plan or task; although this is ideal, it is not always the case. Misbehaviors (errors) such as excessive cauterization or clip placement at the wrong angle is an essential problem because, although it is straightforward to fix errors, it is challenging to eliminate them once they are learned (23).

The apprentice must complete a carefully programmed schedule of training under the instructor's responsibility, who must use debriefing and feedback during advising (27). Debriefing focuses on reviewing, immediately after a real or simulated event, through a discussion among the members who participated in the activity; where actions or responses are reviewed, and the scope of comprehension processes, psychomotor skills, and emotional states to maintain or improve their performance is realized (36). Similarly, feedback is indicated to correct the behavior necessary to learn new skills, through the instructor's respectful suggestion about the behavior (not to the person), to develop specific tasks during training (24, 27). Feedback is formative when it provides detailed information about the skill or behavior while the task is being carried out, regardless if it is being performed correctly or incorrectly, in order to guarantee its adequate understanding and execution. Feedback is summative when it provides information once the training process is accomplished, and the score of training is assigned. Immediate feedback is also considered if you deliver information about the task immediately after the performance is achieved and before it is set in memory. Although it is noted that a large workload can be detrimental to the learner, this type of feedback is associated with better and faster learning and delayed feedback is given once the novice has finished the practice day, which allows the participant to focus their attention on the content of the feedback itself. This type of feedback is associated with better retention over time (24). The best training situations focus on short-term activities with opportunities for verbal, individual and detailed feedback by an expert tutor who observes the learner's performance; also by reflection and immediate corrections of errors or bad behaviors (22, 25). Each completed test should be followed by another similar short task with feedback, until this type of task is completed with constant success (22). Additionally, feedback has been found to serve as a powerful motivator for training (25).

### Configuration of Tasks and Training Strategies in Veterinary Laparoscopic Surgery

The most used central training strategy for the acquisition of psychomotor skills using simulators in laparoscopic surgery is “shaping,” where repetitions are made in successive approximations of the desired task until it is completed according to the desired objective. Tasks can be configured as easy, medium, and difficult, with gradual, smooth, and relatively easy increases in the degree of difficulty (23). The Fundamentals of Laparoscopic Surgery (FLS) is a certified program developed by the American College of Surgeons (ACS) and the Society of American Gastrointestinal and Endoscopic Surgeons (SAGES), for assessment of training in laparoscopic surgery. It represents one of the first validated simulation modules for training physicians and veterinarians. Accomplishing the FLS is a prerequisite for obtaining the board certification of surgery residents in the United States (9, 17, 20), and FLS standards are also applied to surgery fellows and practicing surgeons. In veterinary medicine, there is currently a laparoscopic simulation training program called Veterinary Assessment of Laparoscopic Skills (VALS) created from the Inanimate McGill System for Training and Assessment of Laparoscopic Skills (MISTELS) used by the FLS. The VALS program incorporates task such as pegboard transfer, pattern cutting, ligature loop placement, extracorporeal and intracorporeal suturing, and has been classified as the first training and evaluation program developed for veterinary use (25). The training programs available for laparoscopic surgery were designed for the trainees to develop the basic and advanced skills required for practicing MIS. In addition, training centers and schools of medicine developed protocols for advanced training programs using simulators, where the level of transfer of surgical skills from simulators to real-life (the patient in the surgery room) is controlled (37–39). Basic and advanced training programs have been scheduled, where exercises are sequentially organized in an ascending scale of difficulty, focusing on practicing the technical details of the procedures (26).

Another training strategy, known as “fading,” involves giving learners clues and guides on initial tasks, but as the degree of difficulty increases, the amount of clues and guides gradually fades until the task is done without help. This type of strategy is used by virtual reality simulators (VR) because it has well-defined tasks, as well as instructions and comments integrated into the software, which allow adding or removing elements that guide learning in an automated way (23).

Finally, simulation allows the apprentice to practice the sequencing of learning steps, which is known as task-based simulation. This concept is critical to schedule the training plan; a learning strategy through which a complicated procedure is divided into its basic or simple training units, allowing the repetition and interruption of a task as much as necessary. The novice acquires skills in the individual components before advancing to a more complex task in a clinical setting, decreasing the mental demand that involves carrying out a complex task in a single attempt (32). This strategy has also been presented as a “progressive cumulative experience”; where each student learns a specific task, which he repeats, and receives feedback until achieving the desired competence, then a new task is added, which induces they to continue repeating the first task through the proposed sessions reinforcing and consolidating the surgical skills acquired during the training (37). There is also a pyramid training model for teaching laparoscopic surgery developed at the Jesús Usón Minimally Invasive Surgery Center JUMISC in Spain, which uses this strategy especially at levels 1 (basis of ergonomics and knowledge of instruments and eight exercises for the acquisition of basic skills in laparoscopic surgery), and 2 (surgical techniques in the experimental animal) of the pyramid (39).

Another strategy, very similar to the previous one is known as “backward chaining.” This procedure is divided into discrete psychomotor performance units (task deconstruction) starting from the end of the task, and a new step is added with each step backward or “in chain”; it is used when the apprentice faces difficulties and frustrating tasks to learn (23). In addition to achieving positive results, simulation can lead to wrong behaviors that are difficult to eliminate, so the apprentice must always consider what they want to learn in addition to the expectations guiding the training. In laparoscopic surgery, both strategies have been used successfully, especially for the development of advanced skills such as suturing or even laparoscopic surgical techniques. Unfortunately, there are no known studies in veterinary medicine, where they apply these strategies to learn advanced techniques.

### Training Schedule

After developing the educational curriculum based on theories and learning strategies for training, and with the tasks previously defined, students must practice the task to acquire the proposed skills. The distribution of training time is another critical point in the formation of a training plan. It refers to the frequency required for students to practice the task necessary to acquire simple motor skills. The practice schedule is the most studied variable affecting the acquisition of skills. This refers to the spacing of practice sessions, which could be distributed into long (mass practice) or multiple short practices (interval practice). Although new skills are taught in intensive sessions that often last 1 or 2 days, interval practice is more beneficial for the development of psychomotor skills (24). It is probably because the learned skills require a longer time to achieve cognitive consolidation between practices (23). At the beginning of the acquisition of surgical skills, where the mental demand of the apprentice is high, their mental and physical fatigue interfere with skills training. For that reason, it is recommended to practice a maximum of 1.5 h followed by a break interspersed between sessions, with a maximum of two sessions per day; in this way, learning is consolidated during the resting periods (24). Other authors suggest a distributed practice lasting between 1 and 2 h per day for weeks to months (20). In the case of mass practice, training periods of more than 1 to 2 h per day during short intervals are reported. This schedule can lead to an overestimation of the acquired surgical skills, because memory is not activated before each training, affecting the retention in long-term training (24). The mass practice includes the intensive sessions that often last 1 or 2 days, which sometimes result in learners considering themselves “trained” in this new technology after a short course; therefore, it is recommended that curricula developed for laparoscopic surgical skills training involve distributed interval training. (23).

### Establishment of the Criterion of Competence for Developing Surgical Skills

Currently, training programs in medical education are focused on developing experts in different areas of interest (31). In surgery, there are considerable variations in the learning rate of surgical skills, despite providing the trainees with similar training environments (34). The training time and the number of repetitions to carry out a specific task depends entirely on the level of competence of the apprentice, due to their variable levels of previous skills, innate abilities, and personal motivation (23). Until recently, skill training protocols routinely used arbitrary parameters, such as a total amount of time or multiple repetitions, to determine how much practice should be allowed (20). In veterinary medicine, research in training and evaluation of laparoscopic surgical ability determines the experience of surgeons through previous hospital records as the first assistant surgeon or assistant in laparoscopic procedures, and who certifies more than three years of experience in such procedures before reaching expert status (40).

The number of hours of deliberate practice necessary to reach the expert level is estimated at 10,000 h (25, 36). The experts, people with a high level of competence evaluated objectively, will establish clearly defined objectives of the tasks that the students will use during the training as the required performance criterion at each level before advancing to the next, and thus be able to consider themselves competent. When establishing the criterion of competence, the experts should not be only 1 or 5% of those with superior performance; rather, it should be a representative sample of the competent population. Ideally, national or international benchmarks should be established for competence in simulator performance.

On the other hand, the level of the proficiency criterion should not be too high, because the students will never reach it, and it should not be set too low, because it will produce a set of skills lower than the desired ones (23). Learning curves can allow a better estimate of adequate amounts of practice and identify several repetitions in which a plateau is observed (20, 41). Another critical indicator of training success is consistent performance, because it is not enough to simply improve surgical performance. Since the global objective is to provide surgeons with competent cognitive and psychomotor tools to carry out surgical procedures. One way to further define the competency objective is by evaluating objective skills and training in competency criteria using simulation (23). Competency-based training provides retention of skills of 93% to 99% at five months for basic laparoscopic skills and retention of 90–95% at six months for laparoscopic suturing (20).

Currently, the discussion has focused on trying to carry out a valid selection methodology, with an accurate description of the knowledge, skills, and characteristics necessary to perform in surgery with the idea of identifying students who will acquire skills quickly, minimizing the period of training and thus, guarantee safe and competent professionals in a short time (42). The predictive power of some specific attributes concerning measures of surgical technical capacity (time, errors and efficiency in length and number of movements), have shown that visuospatial perception is correlated with subjective and objective evaluations of surgical performance. A psychomotor aptitude correlates with the rate of acquisition of skills and academic performance predicts the completion of a training program and the passing of exams at the end of said training. Therefore, intermediate and high-level visuospatial perception, as well as psychomotor aptitude, can be used as criteria to evaluate candidates for surgical training (42).

Motivation has also been used as a strategy for students to reach proficiency criteria faster because it is the internal state that activates the teaching and learning process (22). Motivation can be internal or external. Internal motivation is generated by personal interest or satisfaction. Voluntary participation of students responsible for their own schedule and without time allocation is around 6–14%, therefore, it is difficult to carry out a training plan with this type of motivation (25). The construction of clear task objectives, applying reward systems such as participation in real surgeries and the application of serial evaluations that evaluate performance can contribute to this type of motivation. External motivation is developed by the environment in which the student operates and generates effective results in training when compulsory scheduling of sessions is carried out, with flexible hours and with a regulated time. Another aspect that influences external motivation is the level of experience, because it has been determined that the level of experience in laparoscopic surgery is inversely related to the motivation to practice. In addition, training centers that have instructors that actively accompany the learning process and focus on leveling students with deficiencies during training can expect better results in training (22, 25).

### Factors Influencing the Acquisition of Surgical Skill in Veterinary Laparoscopic Surgery

Several reports evidenced that previous experience in console games or video games positively influence the results of training for the acquisition of surgical skill in veterinary laparoscopic surgery. Accordingly, several authors proposed it as a complementary training modality that would serve as a cheap and effective way to improve the simulation for laparoscopic training skills (40, 43–45). Therefore, it has been suggested that manual dexterity is the most critical predictor for achieving appropriate surgical skills (19). Conversely, the level of previous experience in open (non-laparoscopic) surgical procedures (19) and experience in video endoscopic procedures without triangulation (flexible endoscopic procedures) does not influences performance in veterinary MIS (46) (see Table 2).

**Table 2:** Scheduling of training for the acquisition of skills in veterinary laparoscopic surgery.

**Items**	**Characteristics**
Debriefing with good judgment	The instructor creates a context for learning and change in students. Participants openly share their opinion or point of view. Everyone's answers deserve great respect. Errors are discussed in a non-clinical setting.
Feedback	Summative: Basis for making decisions on the level of professional competence of each student (passed/failed, obtaining accreditation or title).
	Formative: Provides immediate information about student learning at the end of a training cycle.
	Immediate: It is delivered immediately after the behavior or error and is done before it is fixed to the memory.
	Delayed: It is delivered once the students have finished their practice or task.
Deliberated practice	Repetitions of tasks outside the clinical setting. Construction of tasks with clearly defined objectives. Constant feedback. Individual motivation to achieve goals.
Training strategies	Shaping: Practice sequences with increasing degrees of difficulty. Fading: Clues and guides on tasks are gradually removed when progressing through difficulty levels. Task-based simulation and Backward chaining: The procedure is divided into simple tasks, and more complex tasks are added, or it begins with the end of the task, and a new step is added backward, respectively.
Distribution of training time	Intensive: a higher number of hours and training in a short period.
	Distributed: high consolidation of information without excessive mental fatigue.
Competition and motivation criteria	Experts establish criteria according to the desired performance and must be achievable. Learning curves estimate the degree of competence. Consistent performance is a good indicator of competition. Internal motivation is generated by personal interest or satisfaction. External motivation is developed by the environment in which the student operates.
Factors that affect the acquisition of surgical skills	Previous experience in console games or video games positively influences Experience in plastic arts positively influences performance in laparoscopic surgery. Previous experience in open surgical procedures (non-laparoscopic) does not influence performance in laparoscopic surgery. Experience in flexible endoscopic procedures does not influence performance in laparoscopic surgery.

## Types of Simulators and Their Technological Complexity

The simulators provide an excellent platform for exerting deliberate practice, which offers the possibility to perform repetitions allowing a training schedule specially designed to improve each individual's performance during the training time (24, 32, 36). Accordingly, the model of competency-based training was implemented since it provides the apprentice with uniform skills independently of the learning curve's characteristics of individual skills acquisition (32). So far, no authors have acknowledged disadvantages in the use of simulation as a method to develop surgical skills, although this fact does not exclude the existence of differences between the simulated model and the surgical exercise in the operating room. Several simulation alternatives are available for surgical training programs in laparoscopic surgery, which could be exerted in animated objects, live animals, or both (21).

A simulator for laparoscopic surgical procedures provides the means for training basic and advanced skills (47), to reproduce the activities of laparoscopic surgery accurately, and face the challenges of the technique and some technical skills not existing in conventional open surgery. These include: (1) Transformation of the three-dimensional surgical space into a two-dimensional video image that affects the perception of depth. (2) The surgeon's hand moves in the opposite direction to that of the instrument on the screen. (3) The fulcrum effect caused by the trocars for accessing the instruments fixed on a point. (4) Laparoscopy reduces the tactile response because it requires long surgical instruments that separate the surgeon's hand of the anatomical structures. And (5) The apprentices tend to ignore their non-dominant hand because of the less direct interaction between the surgeon's hands (28, 34, 48). These facts can reduce the efficiency of laparoscopy, resulting in practicing potentially dangerous surgical procedures when the surgeon is not well-trained (34). The inanimate models are safe, reproducible, portable, easily accessible, and are generally cheaper than animals or corpses (31). Inanimate models include laparoscopic simulation boxes or box models, cadaverous models, and virtual simulators (21).

The simulation boxes for training in laparoscopic surgery (e.g., endotrainers, pelvic trainers, or bench models) were developed shortly after the emergence of laparoscopic surgery due to the need to improve training (34). Although they are used to perform several exercises, such as transference and suture, they lack anatomical precision (avoiding to practice complete procedures) and exhibit low fidelity (21, 31, 34). Their advantages are low cost, high efficiency, rapid implementation, their portable nature, and reproducibility for training basic laparoscopic skills (21, 49). Their design is simple, including a video camera, a monitor, and a box with access points for instruments (34). Several training boxes for humans have been validated for training veterinary surgery, including the following: (1) LapTrainer with SimuVision (Simulab Corp., Seattle, WA, USA) (15, 18, 40, 50); (2) FLS trainer box (SAGES FLS program, Los Angeles, California, USA) (19); and (3) FLS trainer box (Vti medical, Waltham, MA, USA) (40). The Lap Tap Trainer laparoscopy table trainer (3-Dmed, Franklin, Ohio, USA), and T5 Large laparoscopy box trainer (3-Dmed, Franklin, Ohio, USA) training models, were compared to teach students basic skills of laparoscopic surgery. The authors found no differences between training with low-cost simulators or tables compared to a conventional high-cost training box (51). Currently, there are few models of veterinary simulators built and validated for the development of basic and advanced skills for veterinary surgeons. These include the Mayo Endoscopy Simulated Image (MESI) canine abdominal model (Sawbones, Pacific Research Laboratories Inc., Vashon, WA, USA) (17, 18, 50), and Simulvet for laparoscopic training, which was developed from computerized tomography images of three Beagle dogs presented by the Jesus Usón Minimally Invasive Surgery Center (JUMISC) in Cáceres, Spain (14). Both simulators and the training programs proved to have an apparent, constructor, and content validity to learn basic laparoscopic skills (52). A simulator for laparoscopic ovariectomy in standing horses, called standing equine laparoscopic ovariectomy—SELO, was validated by the College of Veterinary Medicine, Washington State University, representing the first specific simulator for this laparoscopic procedure in horses (15).

Virtual reality (VR) and augmented reality (AR) simulators are useful in the acquisition of surgical skills, because they combine performance measures such as time and movement metrics with the possibility of practicing and receiving detailed comments on the exercise quickly. Also, they allow knowing precision measurements, accuracy and error rate (31, 48). Similarly, they offer a potentially unlimited number of procedures on a single platform. However, the technical limitations concerning the perceived realism and the relatively high cost hinder their widespread adoption (20). In veterinary medicine, the use of some of these devices has been reported in research aimed to improve basic skills training, such as the Augmented Reality simulator ProMIS (CAE Healthcare, Montreal, Quebec, Canada) (16, 40). Finally, the virtual reality simulator LapSim (Surgical Science, Minneapolis, Minnesota, USA) (40), and the Lap Mentor medical simulator (3D Systems Healthcare, South Alkire Circle Littleton, CO, USA), are promoted as a complement to laparoscopic surgery training, but augmented reality simulators are more useful for the evaluation of surgical skills than virtual reality ones (40).

In human medicine, human corpses are closer to reality, but their cost, limited availability, and even the deterioration of cadaveric tissue limit their use (31), and specialized centers and trained personnel are required in their preparation and management (49). Generally, the cadaverous models can be of animal or human origin, depending on the procedure to be trained. It has some advantages such as an acceptable fidelity and allowance to achieve a complete simulation of a given surgical technique (21). Finally, the use of live animals is also problematic due to ethical concerns, high costs, and the need for specialized facilities (31). It is important to note that bleeding, hemostasis practicing, workspace restrictions, spatial relationships, visualization of problems, retraction strategies, fan movement artifacts, lighting conditions, and haptic feedback are replicable in live animal models (20), reflecting more closely the conditions of the real-life. (see Table 3).

**Table 3:** Training models available for the acquisition of skills in minimally invasive surgery.

**Simulator**	**Advantages**	**Disadvantages**	**Best use**
Bench model	Economical and portable, reusable, unlimited use allows the apprentice to familiarize with the instruments and pose minimal risks.	Low fidelity; it is not used for basic training-only operations.	They lack anatomical precision, basic surgical skills.
Live animals	High apparent validity in terms of anatomy, haptic ability, availability, can practice hemostasis and complete operations.	Cost, unique facilities, and specialized personnel required, regulations and ethical aspects, different use, anatomical differences of experimental models.	Knowledge of advanced surgical interventions, with the risk of high bleeding, dissection skills.
Cadaveric models	High fidelity, allow practicing full surgeries.	Availability, single-use, regulations, many times more difficult to obtain and more expensive, risk of infection.	Advanced knowledge, dissection, advanced education.
Virtual reality surgical simulator	Reusable, immediate feedback, surgical performance measures.	Expensive, low-perceived realism, not well simulated three dimensions.	Basic laparoscopic skills and procedures.

## Validation and Evaluation Of Surgical Skills in Laparoscopy

### Validation of Surgical Skills

Validation is the process of collecting and interpreting validity evidence (53) and refers to the strength of the evaluation instrument to measure a proposed objective (20). Although some authors conclude that validity only applies to the construct (54, 55), validation of a simulator includes its relevance to provide appropriate task repetition and its reliability to evaluate the acquired surgical skill. Accordingly, it must yield reliable parameters for its evaluation, a quality achieved when the simulator passes a validation process. The classic validity framework is subdivided into five levels: (i) Content validity, which refers to the extent to which all relevant dimensions within a given domain are measured. (ii) Construct validity, which means the reliability to detect differences among groups exhibiting different levels of competence, supporting the principle that the test measures what it says to measure. (iii) Concurrent validity, where it is evaluated the correlation between results of the test with the gold standard criteria used to measure the same domain. (iv) Predictive validity, which is the ability to predict future performance in a different environment. (v) Apparent validity, which is the degree to which the simulation resembles the real task (20, 55). Validity is conceived as a hypothesis, where the collected validity evidence supports hypothesis' acceptance or rejection (56). This type of validation has been performed in human (57) and veterinary surgery (15, 58, 59). Unfortunately, a universally accepted model, including validation as a whole, with no fragmentation of validation levels, does not exist. Recently, a route has been proposed to validate another simulator in laparoscopic surgery that includes the classic validation framework (60).

Currently, it has been suggested that validation is the process of collecting validity evidence to assess the suitability of interpretations, uses, and decisions, based on the results of the simulator evaluation (56). Kane proposed the framework for the inference of validation, where the construct of interest (skills in laparoscopic surgery) and its interpretation (acquisition of laparoscopic skills) are the main goals of validation. Besides, a series of assumptions are scheduled before adopting the most valuable validation instruments, according to the four-components' structured interpretation: punctuation, generalization, extrapolation, and implication, as it was recently approved for MIS. (53, 56). Validation allows reliable judgments and decitions about the apprentice's skills to be made after performing evaluations and provides the strength and limits of the instrument to be understood (56).

### Evaluation of Professional Competencies in Surgery

The educational evaluation assesses several elements of the teaching and learning process necessary to determine the degree to which it contributes to achieving institutional purposes. Therefore, evaluation involves a continuous, systematic, and reflective process through which pertinent qualitative and quantitative information related to a specific objective is acquired. It allows the identification of the strengths and areas of opportunity in order to make a judgment that approves fundamental decision making for the improvement of the evaluated activity (54).

Traditionally, the training model for learning surgical skills is carried out under the classic doctrine of “seeing, doing, and teaching,” which implies practicing on the patient with a subjective evaluation made under direct observation and entirely dependent on the tutor (20, 61). This approach is not adequate for evaluating training in laparoscopic surgery, due to the technical differences it has compared to conventional surgery (20, 39). The methods used to evaluate surgical ability in open surgery training programs are not reliable in laparoscopic surgery, because there are no error tracking systems and they lack integrated timers (20). One option is the Kirkpatrick model that focuses on the assessment by learning levels: Level 1 or reaction, which evaluates the reaction of participants in the training program, seeking the degree of apprentices' satisfaction. Level 2 or learning, which focuses on the evaluation of acquired competencies. Level 3 or behavior, where it evaluates the transfer of learning to real–life. Level 4 or results, which assesses the impact of training on society or the population of influence (62).

Miller's model is another method of evaluating laparoscopic surgical competence developed in 1990. It is currently the most widely accepted method of evaluating professional competence in medical education. This was built based on the Kirkpatrick model and conceptualizes the levels of professional competence in a pyramid form (called Miller's pyramid). From the base to the top includes: Level 1 or the base, which evaluates knowledge, tested with oral or written exams. Level 2 or “know-how” (application of knowledge), which includes, in addition to level 1, the cognitive aspect of competence and contains skills such as decision making and clinical reasoning. Level 3 or “demonstrate how,” where the evaluation of clinical competence is qualitative and contains the skills applied in a non-real context (demonstrates how it does it in simulated contexts). Level 4 or vertex, which evaluates the “doing” (action), meaning the evaluation of competences demonstrated under real-life situations (62).

Considering the objective of the evaluation, it can be diagnostic, formative, and summative (54). (i) diagnostic evaluation, allows to determine the progress of the student in the acquisition of knowledge, skills, attitudes, and values that allow you to go to the next level or repeat the task; likewise, it serves to identify failures or strengths. (ii) formative evaluation, which provides permanent and progressive information on learning, reinforces weaknesses by establishing corrective strategies and improvements in the process. Finally, (iii) the summative evaluation, allows the evaluation of the learning and certifies that the students have reached the competence they intend to acquire (54, 62).

There are different instruments for evaluating laparoscopic surgical skills that include scoring systems for skill training, that can reveal the progression of surgical skill and mistakes. Hence, the instruments for evaluating the training device, simulators, or curriculum must meet a series of criteria, supporting the validity and reliability of the process (21, 28, 54). Reliability is defined as the ability of the evaluation schedule and device to produce a consistent result when it is repeated. In this context, the reproducibility of the test and the accuracy of a device. The characteristic of making a simulator reliable is its robustness and durability for allowing constant practice and repeatability of results, which are evaluated by a coefficient ranging from 0.0 to 1.0 (no correlation to perfect correlation, respectively). The result of reliability between R = 0.5 and 0.8 is moderate, and > 0.8 is high (20, 63). Other authors define the correlation as: R = 0.8 to 1, excellent correlation; R between 0.5 and 0.79, good correlation; R between 0 and 0.5, poor correlation; and R between 0 and−1, disagreement (64).

## Motion Capture Devices as an Instrument to Assess Surgical Skills in Laparoscopic Surgery

The instruments available for training in laparoscopic surgery have evolved into devices that include efficient data management systems, both in box-trainer simulators and virtual reality simulators that can be used to perform a debriefing assessment or evaluation. The measures include instrument movements, limb movements, eye movements, tension applied to the simulated tissues, and knots constructed (20). One of the first devices designed to use tracking technology was the Dundee Advanced Psychomotor Tester (ADEPT), designed at the University of Dundee (Dundee, UK) (20, 61, 65, 66). The ADEPT records the time, range of movement of the shoulder by sensors of three-dimensional movement, and safety of the knot through a tensiometer, using sensors in the dominant arm. Although studies showed construct validities, its application is not widespread (65).

Another method is a measurement of laparoscopic instrument movements with electronic sensors designed to measure the economy of movements, expressed as the distance elapsed by the instrument or the sum of the deviations from a designated focal point. The resulting data indicates the degree of dexterity, operational focus, and overall experience. An example of this technology is the Skills Assessment Device (SAD) designed at Emory University (Atlanta, USA) (20, 67). Motion sensors have also been used to track the surgeon's hands during the manipulation of surgical instruments. A study by the Southwestern Medical Center at the University of Texas examined the impact on movement economy between the use of a robot-assisted camera (RACC) in front of an expert human camera operator, using motion sensors initially placed both on the hands of the surgeon and on the laparoscopic camera. In this study, no significant differences were found between operative times or hand movements. Although the accumulated rotary movement was increased with a skilled human operator compared to the robot, the surgeon could work with the same efficiency with an almost ideal image (20, 68).

Training tasks with a computer interface have also been used to create a scoring system independent of the task, which is compared with the performance of experts, measuring time, road length, smoothness of movement, the perception of depth, and orientation. This device, that was designed between the Boston Center for the Integration of Medicine and Innovative Technology (CIMIT) and Harvard Medical School, was called Computer-Enhanced Laparoscopic Training System (CELTS). This interface was classified as a sophisticated method with simple metrics that may not represent a measure of significant competence (20, 69) and has demonstrated a reasonable apparent validity through surveys, with limited construct validity (61). The Faculty of Medicine of the Imperial College of London validated an electromagnetic motion tracking system called Imperial College Surgical Assessment Device (ICSAD), designed to evaluate hand movements during surgical exercises in laparoscopic surgery. In ICSAD, sensors are placed on the back of the hand and the third finger when developing a task (20, 31, 66, 70). It measures the time spent, the number of movements, and path-length. Although it is a valid and reliable instrument for evaluating laparoscopic skills, exhibiting good agreement with the OSATS score (31), other authors consider that the validity of the construct is limited to specific tasks (54).

Lemos et al. designed from the Faculty of Engineering, University of Antioquia (Medellin, Colombia) designed a hand glove-based portable device that uses inertial motion units (IMU) for the evaluation of manual dexterity in simulation-based neurosurgical education. The “I-Glove” was designed for its use in the Daubara NS Trainer neurosurgical simulator (71), although it could be used in other medical simulators based on test benches and mannequins. Currently, this device is being validated by a comparison between the “I-Glove” and the LapSim metric evaluation device (71). The Faculty of Medicine of the Imperial College of London, recently implemented the use of a new system that is based on monitoring eye movements, specifically the visual accommodation (tonic, or pupil visual adaptation), which is a new metric for surgery that has been assessed in aircraft pilots. This type of technology evaluates how surgeons could acquire and react to visual stimuli from a flat monitor (20, 61).

In veterinary medicine, the use of tools for the evaluation of hand movement during training in basic tasks such as CyberGlove Systems LLC (San Jose, California), has been implemented. It is composed of 16 sensors with resistance flows that are sensitive to finger and hand flexion and was used to evaluate hand movements in veterinarians who performed laparoscopic training with basic tasks (59). Likewise, commercial AR and VR training devices that incorporate movement evaluation technology, have been used in humans to validate evaluation methods or during basic skills training (40). The validity of movement analysis used to evaluate laparoscopic ability most frequently measure time, length of movement trajectory, and some hand movements. However, because surgical competence is multimodal, the implementation of movement analysis as the only measure for the specific assessment of manual dexterity isolates the apprentice from the context of the operating room. Therefore, it is recommended to evaluate together with motion capture systems and with global and specific scales that allow evaluating the precision of the task and the results (66) (see Table 4).

**Table 4:** Evaluation instruments used for training schedules in minimally invasive surgery.

**Instrument**	**Characteristics**	**Ref**.
The advanced psychomotor tester of Dundee	It is an objective real-time scoring system designed by the University of Dundee, Nethergate, Dundee, Scotland, UK, which corrects the subjective opinion of the advisor in the execution of endoscopic tasks.	(65)
Skill Assessment Device (SAD)	It measures movements at the tip of the instruments designed by Emory University School of Medicine, Atlanta, GA, USA.	(67)
Robot-assisted camera control (RACC)	This instrument is valid and reliable for intraoperative evaluation and provides formative and summative comments of participants.	(68)
Computer-enhanced laparoscopic training system (CELTS)	It is a computer-based system designed by the Simulation Group Center for Integration of Medicine and Innovative Technology (CIMIT), the USA, capable of tracking the motion of laparoscopic instruments and providing real-time feedback about performance.	(69)
Imperial College surgical assessment device (ICSAD)	This device was created in the Imperial College London (London, UK). The ICSAD system consists of an electromagnetic tracking system (Isotrak II, Polhemus Inc., Colchester, VT) connected to a laptop with an independent motion acquisition software and custom analysis software to convert position data into the dexterity measures.	(70)
Cyberglove	It was created by the company CyberGlove Systems LLC in San Jose, California. This device captures up to 22 measurements of hand and finger movements. The number of sensors depends on the reference. The glove has flexion sensors per finger in the curvature on each finger, abduction sensors, sensors measuring the crossing of the thumb, the arch of the palm, the flexion of the wrist and the abduction of the wrist	(59)
IGlove	The portable device created by Bioinstrumentation and Clinical Engineering Research Group-GIBIC, Bioengineering Department, Engineering Faculty, University of Antioquia, Medellin, Colombia, that uses inertial sensors embedded in an elastic glove for recording hand movements. It provides data on time and a wide range of movements.	(71)

## Objective Structured Evaluation as an Instrument to Evaluate Surgical Ability in Laparoscopic Surgery

The assessment of technical performance by expert observers (coaches) remains an essential tool for evaluation, which can be done orally or by writing a report (20). The evaluation of valid and reliable technical skills is needed in order to plan the tasks to be performed and evaluated during training schedules (31), and includes global rating scales, specific classification scales, and analysis of task errors (20). Previously, surgical ability was assessed using In-Training Evaluation Reports (ITERs) performed directly by the assistant surgeon. Competence and learning curve were built from the number of procedures performed in a certain period of time. However, ITERs exhibits bias of central tendency, cognitive bias (”halo effect"), and memory bias (34). The evaluation of cognitive and psychomotor skills is difficult within the operating room, the apprentice is moved to settings outside the operating room under the direct observation of an expert. The first studies designed for evaluation of surgical skills were focused on documenting the improvement in technical skills after practicing in the simulator and used the same simulation platform to perform the evaluation. Therefore, there is a persistent gap in transferability from the simulator to the clinical or non-simulated settings (20).

One of the curricula most commonly used in veterinary laparoscopic surgery is the MISTELS program, that has been developed and validated for training and evaluation of a set of five basic surgical psychomotor skills (18, 31, 50). Its main disadvantage is that the evaluations are limited to execution time and task errors (40).

The Objective Structured Assessment of Technical Skills (OSATS) scale is one of the most cited scales because of its moderate-to-high reliability and acceptable construct validity (31, 34). The evaluation methods for OSATS include a global rating scale (GRS) and a specific rating scale (SRS) or task-specific checklist. GRS includes five to eight measurements of standardized surgical procedures applied to inanimate models, each qualifying from 1 to 5 with a Likert scale. The topics evaluated include (i) respect for tissue, (ii) time and movement, (iii) handling and knowledge of instruments, (iv) use of assistants, (v) flow of operation (advanced planning), and (vi) knowledge of the procedure (20, 34). It is noteworthy that OSATS was not developed for the evaluation of laparoscopic skills and did not provide evidence to demonstrate the transferability of skills from laparoscopic simulators to the operating room (34). This limitation resulted in the development of the global operative assessment of laparoscopic skills (GOALS) scale at McGill University in 2005, based on Reznick's OSATS tool specifically designed for minimally invasive procedures (20, 72). Currently, not only has it been promoted for the evaluation of ability in laparoscopic cholecystectomy, its use in other laparoscopic procedures has also been validated (72). The GOALS scale scores from 1 to 5 with a Likert scale, and includes the evaluation of skills such as depth perception, bimanual dexterity, efficiency, tissue management, autonomy, and difficulty level of the task to be performed (20) (see Table 5).

**Table 5:** The scale of qualification and assessment of laparoscopic skills in human and veterinary medicine.

**Skill domain**	**Punctuation**				
	**1**	**2**	**3**	**4**	**5**
Depth perception	It consistently exceeds the objective, has large oscillations; it requires a long time to correct.		Has some overshoots or lack of objective, is quick to correct.		Directly guide the instruments in the correct plane to the objective.
Bimanual dexterity	Just use one hand and ignore the non-dominant hand, weak hand coordination.		Use both hands, but do not optimize the interactions between both.		Use both hands expertly, and the movements are complementary, optimizing the exposure.
Efficiency	Uncertain movements, inefficient, ineffective or tentative efforts, continually changing, blurring of the objective of the task, persisting without progress.		Slow but planned and reasonably well-organized movements.		Confident and efficient with a safe behavior, maintain the focus on the task until it develops appropriately.
Tissue handling	Sudden movements with tissue tearing, excessive traction, injuries in adjacent structures, poor grip control that often slips, poor control of coagulation device.		Handles tissues reasonably well, occasional sliding of the forceps, occasionally causes bleeding, minor trauma to adjacent tissues with the energy source.		Handles tissues well with adequate traction, insignificant injuries in adjacent structures, and efficient use of energy sources.
Autonomy	Apprentice cannot complete assigned tasks, even under verbal prompts.		Able to complete the assigned tasks with moderate indications.		Able to complete assigned tasks independently, probably without warning or help.
Level of difficulty	Easy exploration and dissection.		Moderate difficulty (scarring, adhesions, somewhat inferior tissue planes).		Extremely difficult (scarring, adhesions, inferior tissue planes).

*Source: Modified from Vassiliou et al. (72)*.

**Figure 2:**
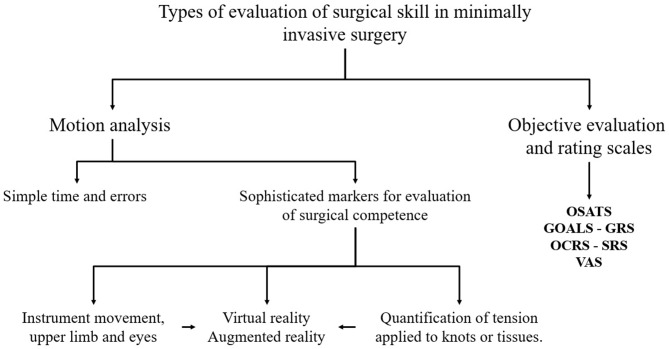
Schematic diagram of the methods to evaluate the acquisition of surgical skills in training for minimally invasive surgery. Objective Structured Assessment of Technical Skills (OSATS), Global operative assessment of laparoscopic skills (GOALS), Global rating scale (RGS), Specific rating scale (SRS), Operational component rating scale (OCRS), Visual analog scales (VAS).

The operational component rating scale (OCRS) is a specific qualification form for a surgical procedure, which is segmented into its simplest components to be evaluated individually regarding the technical ability of each student on a 5-point Likert scale or Visual analog scales (VAS). For this scale, experts reach a consensus on what steps or tasks should be taught to the apprentice, in order to facilitate learning and evaluation of the proposed surgical technique (18). Specific rating scale (SRS) have been used for the evaluation of laparoscopic skills that have previously been considered essential elements of the procedure to be taught in a program of MIS training (52) (see Figure 2). Generally, this scale should be previously validated by experts and a Likert-type scale or the checklist (done = 1 point, not done = 0) can be used for the rating. The main disadvantage of the scale is that the checklists consist of yes or no questions, related to specific elements of an evaluated procedure, which makes it rigid and inadmissible for an integral evaluation (72).

VAS are used to assess the surgical skill or performance for the different tasks assigned in training, with grades ranging from 0 millimeters (mm) to 100 mm, where 0 mm means that the task is not completed and 100 mm means that the task is done correctly. It is also used to rate other types of evaluation methods (18). Additionally, some validation work has been used to determine the level of experience of the participants, where 0 is considered inexperienced, 50 mm is considered with little experience according to the number of procedures that have been performed, and 100 mm is considered an expert by its academic level and number of interventions. In the same way, it can be done with the experience of console games (17) (see Table 6).

**Table 6:** Scoring systems for the acquisition of skills in minimally invasive surgery.

**Scale**	**Characteristics**
Objective structured assessment of technical skill-OSATS	Developed in the '90s in the University of Toronto, Canada, at present, it is one of the most used to teach and to evaluate abilities in the medical practice. It is typically used to evaluate several medical specialties—for example, conventional surgery, nursing, anesthesia.
Global assessment of laparoscopic skill-GOALS	It was created at the Centre for Minimally Invasive Surgery in McGill University, USA, for assessment of intraoperative laparoscopic skills. It allows the evaluator to incorporate their experience to improve student performance and take advantage of the evaluation for teaching; this is based on the balance of GOALS, in terms of structure and objectivity.
Operative component rating scale (OCRS)	It is a specific qualification record of the operation that evaluates each task of the particular procedure being evaluated. It is developed by a consensus defining the critical tasks of the procedure. The model is evaluated by Likert or VAS scales.
Specific rating scale (SRS)	This scale is constructed considering the evaluator's objectives, although focused on what is essential for the student to learn during training. Usually, these scales are previously validated with experts and typically qualify with Likert scales.
Task-specific checklists	It consists of yes and no questions about specific components of a procedure.
Visual Analog scale (VAS)	This evaluation does not provide enough structure and is subjective. The evaluator places a mark (X) indicating the score assigned for the particular task. The scale is scored on a horizontal line starting at 0 (cm or mm), representing the absence of the activity to be developed or any other statement that indicates if the task was not accomplished. The scaling limit finishing at 10 cm or 100 mm, representing the satisfactory or optimum development of the task to be performed.
Cumulative sum (CUSUM) analysis	It is applied to surgical procedures, considering if the surgical intervention is successful or failed, and the rate of acceptance of complications is evaluated. The analysis yields a jagged curve, which once flattened, and with decreasing scores, indicates success. On the contrary, if the score increases, it indicates that errors or complications persist while performing the task.

Some of the general limitations of assessment using scales are associated with the need for expert direct observers and the costs this entails during training to assess student movements and errors; therefore this limits widespread use of training (20). This limitation is similar for the evaluation through video records that contain the sequence of tasks carried out by the apprentices. Both edited and unedited video does not correlate with direct observation and has low reliability among evaluators. This is possibly because the video does not provide additional information such as the audio or image of the external environment that is crucial to understand some of the movements carried out in the tasks (73).

Even though we are aware of the usefulness of measurement scales for training and evaluation of the surgical skill in veterinary medicine, we propose three punctual modifications to the GOALS. First: The handling of devices (suture, electrosurgery, vascular sealing, and stapling). Although the scale includes an item for instrument handling, the definitions in the items do not allow the evaluation of such devices. Besides, the use of pedals could affect operational performance (74); therefore, there could be evaluation bias. Second: The introduction and extraction of anatomical materials and pieces, which is an item that has many evaluating methods, resulting in a lack of unified criteria on the correct form of pieces' extraction within the cavities. Third: The entrance of the first port or establishment of the pneumoperitoneum, there are no known didactic means (simulator) for training or a study plan where the apprentice can practice and be evaluated. Curiously, the vast majority of complications in veterinary laparoscopic surgery are due to the wrong performance of this fundamental task (58, 75) (see Table 7).

**Table 7:** Additional items proposed to modify the global operational assessment scale of laparoscopic skills (GOALS).

**Skill domain**	**Punctuation**				
	**1**	**2**	**3**	**4**	**5**
Introduction and extraction of anatomical materials and parts	Try to introduce sutures with large needles through small trocars or improperly, take out the trocar to insert a piece, do not use bags for the extraction of small items. It does not provide for the use of a large-sized port for the introduction or extraction of predictable elements.		Try to introduce sutures with large needles through small trocars or improperly, but quickly reconsider, use the percutaneous route in individual cases, occasionally take out the trocar to insert a piece, use the bags for the extraction of small-sized elements. It provides for the use of a large-sized port for the introduction or extraction of predictable elements.		Enter trocar suture according to size and taking into account the needle. Use the percutaneous route in individual cases, keep the trocars in their initial position, quickly reintroduce small items into bags for extraction. It provides for the use of a large-sized port for the introduction or extraction of predictable elements.
Handling of devices (suture, electrosurgery, sealing, stapling)	It becomes entangled with the suture, loses sight of the needle, burns the tissue too much at different points until it is carbonized or/and does not respond to the warning of the sealing device, hesitates to locate a clip, places tiny clips or does not take into account the size of the structure to be sealed or one over another.		It gets entangled a little without losing the course of the task that must be done, does not lose sight of the needle, cauterizes just enough to cut, but at different points and heeds the alert of the sealing device, places the clips according to the anatomical structure one above the other or very close to the cut.		It is developed correctly with the suture inside the surgical space without losing sight of the needle, cauterizes just enough to cut or hear the alert of the sealing device, places the clips according to the anatomical structure correctly.
The entry of the first port or establishment of the pneumoperitoneum	Veress technique (closed): It does not make an incision of the skin; it takes the needle interchangeably near the key. Insert the needle too hard and obliquely. Hasson techniques (open or mini-laparotomy): does not measure the size of the trocar incision, make a full-thickness incision without disclosure. Do not place the other ports under direct vision.		Veress technique (closed): Make an incision of the skin, take the needle interchangeably near the key or in the body. Insert the needle gently and obliquely. Hasson techniques (open or mini-laparotomy): Measure the size of the trocar incision, make a full-thickness incision without disclosure. Place the other ports with the direct vision into space without looking at the screen.		Veress technique (closed): Makes a small skin incision, takes the needle 1 to 2 centimeters near the tip, gently introduces rotational movement, and the needle perpendicularly. Hasson techniques (open or mini-laparotomy): Pre-measure the size of the trocar incision, make skin incision and enter with blunt spreading with a mosquito. Place the other ports under direct vision.
Feedback Comments					

Finally, the cumulative sum technique (CUSUM) is an alternative for the evaluation of the surgical performance of surgeons, compared to a previously defined standard performance. This allows us to graph the learning curve of an assigned task and control the increase in complication rates in a surgical procedure. In a study conducted by Pope (2014), the author demonstrated the usefulness of the CUSUM technique in determining that ~80 laparoscopic ovariectomies are needed in bitches in order to achieve a minimal level of competence (41).

## Conclusion

The most common teaching method for laparoscopic surgery in veterinary medicine is still the traditional method, which consists of “observing, helping, and operating.” However, the use of validated training plans using simulators, provide an essential tool to evaluate the acquisition of surgical skills, a fact that is challenging for the training of veterinary surgeons (52), in as much as traditional training demands more time compared to laparoscopic training with simulators (41). In veterinary MIS, there is a lack of curricula for the training of advanced skills and specific simulation models for advanced techniques that makes it difficult to separate the practice of surgical techniques on experimental animals or cadavers. The learning curve will depend on tutoring time by an expert, incidence of clinical cases (disease) of interest during training, level of experience, and availability of a tutor who wants to teach. Similarly, learning this technique, itself, is more complicated than open surgery (21). In addition to its high cost, legal limitations, ethical and current legislation restricting the use of animals for *in-vivo* practices, the use of advanced techniques for training is restricted to immediate use, with no opportunity to perform deliberate practice (21).

On the other hand, the principle of not negatively affecting the patient's safety is the most critical factor limiting training in the real patient, which could threaten the life of patients due to intraoperative complications such as splenic lacerations, perforations of the urinary bladder and subcutaneous emphysema which are the most common surgical errors in dogs, and which are dependent on the training curve (41, 75). Causing a laceration or perforation of a vital organ during training would result in a change of the procedure to a laparotomy, which would very likely result in probable consequences for the patient as well as ethical implications for the apprentice surgeon. From a rational point of view, it is problematic to continue to complete the learning curve on patients, as is currently done in many training settings (21). Training in MIS requires the application of tools such as cumulative sum analysis that allow us to track progress/results in simulated and real environments in a more orderly and efficient way (41). Finally, the achievement of necessary skills for laparoscopy, according to FLS parameters, are generally the same as those developed for both human and veterinary surgeons, so it would not be a disadvantage to develop them at national or international training centers. The primary limitations of training MIS in veterinary surgery include the lack of optimal programs to develop advanced skills in a particular surgical technique, which use assessment methods such as motion capture devices with user-friendly software that allows students to self-evaluate their performance. In general, we can assess the surgical capacity of a student using inertial or electromagnetic devices to record movements or the method using scales that require direct or indirect observation by an expert. However, it will be preferred to use them together because they are complementary evaluation methods.

Similarly, the objective evaluation scales as a complement to ensure meaningful learning of students interested in MIS and the current information on training in veterinary medicine is scarce (17). To achieve advanced laparoscopic skills in veterinary medicine, it is necessary to establish simulation programs compromising simulators and curricula. These programs must be validated with a higher number of evaluations, and their effectiveness must guarantee to the veterinary surgeons interested in learning specific techniques, the optimal acquisition of expert-level required to develop an advanced surgical technique.

## Author Contributions

CO-P conceived the review and wrote the original version of the manuscript. AT-A, JC, JL, and CR-B reviewed, and critically contributed to improving the content of the manuscript. JM-E reviewed and corrected the manuscript and verified the final version with CO-P. All authors agreed and approved the final version of the manuscript.

## Conflict of Interest

The authors declare that the research was conducted in the absence of any commercial or financial relationships that could be construed as a potential conflict of interest.

## References

[1] Matilla JR Klepetko W Moser B. Thymic minimally invasive surgery: state of the art across the world—Europe. J Vis Surg. (2017) 3:70. 10.21037/jovs.2017.04.01 29078633 PMC5637941

[2] Wong MKH Sit AKY Au TWK. Minimally invasive thoracic surgery: beyond surgical access. J Thorac Dis. (2018) 10:S1884–S91. 10.21037/jtd.2018.05.196 30026975 PMC6035932

[3] Baukloh J-K Perez D Reeh M Biebl M Izbicki JR Pratschke J. Lower gastrointestinal surgery: robotic surgery versus laparoscopic procedures. Visc Med. (2018) 34:16–22. 10.1159/000486008 29594165 PMC5869491

[4] Mayhew PD. Recent advances in soft tissue minimally invasive surgery. J Small Anim Pract. (2014) 55:75–83. 10.1111/jsap.12164 24372087

[5] Hettlich BF. Minimally invasive spine surgery in small animals. Vet Clin North Am Small Anim Pract. (2018) 48:153–68. 10.1016/j.cvsm.2017.08.008 29037433

[6] Milovancev M Townsend KL. Current concepts in minimally invasive surgery of the abdomen. Vet Clin North Am Small Anim Pract. (2015) 45:507–22. 10.1016/j.cvsm.2015.01.004 25758850

[7] Cléroux A. Minimally invasive management of uroliths in cats and dogs. Vet Clin North Am Small Anim Pract. (2018) 48:875–89. 10.1016/j.cvsm.2018.05.008 30098647

[8] Tapia-Araya AE Martin-Portugués ID-G Sánchez-Margallo FM Veterinary laparoscopy and minimally invasive surgery. Companion Anim. (2015) 20:382–92. 10.12968/coan.2015.20.7.382

[9] Fransson BA Mayhew PD editors. Small Animal Laparoscopy and Thoracoscopy. Ames, IA: Wiley (2015).

[10] Tapia-Araya AE Díaz-Güemes Martin-Portugués I Fresno Bermejo L Sánchez-Margallo FM. Laparoscopic ovariectomy in dogs: comparison between laparoendoscopic single-site and three-portal access. J Vet Sci. (2015) 16:525. 10.4142/jvs.2015.16.4.525 26119164 PMC4701746

[11] Devitt CM Cox RE Hailey JJ. Duration, complications, stress, and pain of open ovariohysterectomy versus a simple method of laparoscopic-assisted ovariohysterectomy in dogs. J Am Vet Med Assoc. (2005) 227:921–7. 10.2460/javma.2005.227.921 16190590

[12] McCarthy TC Constantinescu GM Veterinary Endoscopy for the Small Animal Practitioner. St. Louis, Mo: Elsevier Saunders (2005).

[13] Oviedo-Peñata CA Hernández López CA Laparoscopy versus parapreputial laparotomy for the treatment of abdominal cryptorchidism in dogs. CES Med Vet Zootec. (2013) 8:83–92.

[14] Usón-Gargallo J Tapia-Araya AE Díaz-Güemes Martin-Portugués I Sánchez-Margallo FM. Development and evaluation of a canine laparoscopic simulator for veterinary clinical training. J Vet Med Educ. (2014) 41:218–24. 10.3138/jvme.0913-136R1 25000884

[15] Elarbi MM Ragle CA Fransson BA Farnsworth KD. Face, construct, and concurrent validity of a simulation model for laparoscopic ovariectomy in standing horses. J Am Vet Med Assoc. (2018) 253:92–100. 10.2460/javma.253.1.92 29911940

[16] Lencioni RD Ragle CA Kinser ML Coffey T Fransson BA. Effect of simulator orientation during skills training on performance of basic laparoscopic tasks by veterinary students. J Am Vet Med Assoc. (2017) 251:1196–201. 10.2460/javma.251.10.1196 29099249

[17] Chen C-Y Ragle CA Lencioni R Fransson BA. Comparison of 2 training programs for basic laparoscopic skills and simulated surgery performance in veterinary students. Vet Surg. (2017) 46:1187–97. 10.1111/vsu.12729 28990691

[18] Fransson BA Ragle CA Bryan ME. A laparoscopic surgical skills assessment tool for veterinarians. J Vet Med Educ. (2010) 37:304–13. 10.3138/jvme.37.3.304 20847341

[19] Kilkenny JJ Singh A Kerr CL Khosa DK Fransson BA. Factors associated with simulator-assessed laparoscopic surgical skills of veterinary students. J Am Vet Med Assoc. (2017) 250:1308–15. 10.2460/javma.250.11.1308 28509639

[20] Tsuda S Scott D Doyle J Jones DB Surgical skills training and simulation. Curr Probl Surg. (2009) 46:271–370. 10.1067/j.cpsurg.2008.12.003 19249439

[21] León Ferrufino F Varas Cohen J Buckel Schaffner E Crovari Eulufi F Pimentel Müller F Martínez Castillo J Simulación en cirugía laparoscópica. Cir Esp. (2015) 93:4–11. 10.1016/j.ciresp.2014.02.011 25039039

[22] Anders Ericsson K. Deliberate practice and acquisition of expert performance: a general overview. Acad Emerg Med. (2008) 15:988–94. 10.1111/j.1553-2712.2008.00227.x 18778378

[23] Gallagher AG Ritter EM Champion H Higgins G Fried MP Moses G. Virtual reality simulation for the operating room: proficiency-based training as a paradigm shift in surgical skills training. Ann Surg. (2005) 241:364–72. 10.1097/01.sla.0000151982.85062.80 15650649 PMC1356924

[24] Opazo Morales EI Rojo E Maestre JM Modalidades de formación de instructores en simulación clínica: el papel de una estancia o pasantía. Educ Médica. (2017) 18:22–9. 10.1016/j.edumed.2016.07.008

[25] Fransson BA. Advances in laparoscopic skills training and management. Vet Clin North Am Small Anim Pract. (2016) 46:1–2. 10.1016/j.cvsm.2015.08.002 26396055

[26] Camacho FJ Herrrera DP Peralta MC Rodríguez GP Cortéz M Alonso GO F. Estandarización de un nuevo método de entrenamiento para la adquisición de habilidades en cirugía endoscópica mediante el empleo de simuladores quirúrgicos. RevMedicaSanitas. (2010) 13:40–5.

[27] Ruiz-Gómez JL Martín-Parra JI González-Noriega M Redondo-Figuero CG Manuel-Palazuelos JC. La simulación como modelo de enseñanza en cirugía. Cir Esp. (2018) 96:12–7. 10.1016/j.ciresp.2017.09.005 29054573

[28] Kerrigan N Simulación, ¿una necesidad en el entrenamiento para la cirugía laparoscópica colorrectal? Rev Chil Cir. (2017) 69:508–12. 10.1016/j.rchic.2017.06.004

[29] Russell JA Barrett LF. Core affect, prototypical emotional episodes, and other things called emotion: dissecting the elephant. J Pers Soc Psychol. (1999) 76:805–19. 10.1037//0022-3514.76.5.805 10353204

[30] Kneebone R. Evaluating clinical simulations for learning procedural skills: a theory-based approach. Acad Med J Assoc Am Med Coll. (2005) 80:549–53. 10.1097/00001888-200506000-00006 15917357

[31] Reznick RK MacRae H. Teaching surgical skills — changes in the wind. N Engl J Med. (2006) 355:2664–9. 10.1056/NEJMra054785 17182991

[32] Kolozsvari NO Feldman LS Vassiliou MC Demyttenaere S Hoover ML. Sim one, do one, teach one: considerations in designing training curricula for surgical simulation. J Surg Educ. (2011) 68:421–7. 10.1016/j.jsurg.2011.03.010 21821224

[33] Tenison C Anderson JR. Modeling the distinct phases of skill acquisition. J Exp Psychol Learn Mem Cogn. (2016) 42:749–67. 10.1037/xlm0000204 26551626

[34] Choy I Okrainec A. Simulation in surgery: perfecting the practice. Surg Clin North Am. (2010) 90:457–73. 10.1016/j.suc.2010.02.011 20497820

[35] Oropesa I Sánchez-González P Lamata P Chmarra MK Pagador JB Sánchez-Margallo JA. Methods and tools for objective assessment of psychomotor skills in laparoscopic surgery. J Surg Res. (2011) 171:e81–e95. 10.1016/j.jss.2011.06.034 21924741

[36] Maestre JM Rudolph JW Teorías y estilos de debriefing: el método con buen juicio como herramienta de evaluación formativa en salud. Rev Esp Cardiol. (2015) 68:282–5. 10.1016/j.recesp.2014.05.018 25239179

[37] Varas J Mejía R Riquelme A Maluenda F Buckel E Salinas J Significant transfer of surgical skills obtained with an advanced laparoscopic training program to a laparoscopic jejunojejunostomy in a live porcine model: feasibility of learning advanced laparoscopy in a general surgery residency. Surg Endosc. (2012) 26:3486–94. 10.1007/s00464-012-2391-4 22733192

[38] Cáceres-Ferro D Cortés-Barré M Desarrollo de un procedimiento quirúrgico aplicable como programa de entrenamiento en cirugía. Revista Colombiana de Obstetricia y Ginecología. (2013) 64:107–14. 10.18597/rcog.117

[39] Usón-Gargallo J Pérez-Merino EM Usón-Casaús JM Sánchez-Fernández J Sánchez-Margallo FM. Modelo de formación piramidal para la enseñanza de cirugía laparoscópica. Cir Cir. (2013) 81:420–30. 25125060

[40] Fransson BA Chen C-Y Noyes JA Ragle CA. Instrument motion metrics for laparoscopic skills assessment in virtual reality and augmented reality: motion metrics for laparoscopic skills assessment. Vet Surg. (2016) 45:O5–O13. 10.1111/vsu.12483 27239013

[41] Pope JFA Knowles TG. Retrospective analysis of the learning curve associated with laparoscopic ovariectomy in dogs and associated perioperative complication rates: learning curve associated with laparoscopic ovariectomy in dogs. Vet Surg. (2014) 43:668–677. 10.1111/j.1532-950X.2014.12216.x 24962374

[42] Maan ZN Maan IN Darzi AW Aggarwal R. Systematic review of predictors of surgical performance. Br J Surg. (2012) 99:1610–21. 10.1002/bjs.8893 23034658

[43] Millard HAT Millard RP Constable PD Freeman LJ. Relationships among video gaming proficiency and spatial orientation, laparoscopic, and traditional surgical skills of third-year veterinary students. J Am Vet Med Assoc. (2014) 244:357–62. 10.2460/javma.244.3.357 24432969

[44] MacCormick MRA Kilkenny JJ Walker M zur Linden A Singh A. Investigating the impact of innate dexterity skills and visuospatial aptitude on the performance of baseline laparoscopic skills in veterinary students. Vet Surg. (2017) 46:1175–86. 10.1111/vsu.12682 28892186

[45] Bragg HR Towle Millard HA Millard RP Constable PD Freeman LJ. Association of gender and specialty interest with video-gaming, three-dimensional spatial analysis, and entry-level laparoscopic skills in third-year veterinary students. J Am Vet Med Assoc. (2016) 248:1414–8. 10.2460/javma.248.12.1414 27270065

[46] Fransson BA Ragle CA Bryan ME. Effects of two training curricula on basic laparoscopic skills and surgical performance among veterinarians. J Am Vet Med Assoc. (2012) 241:451–60. 10.2460/javma.241.4.451 22852570

[47] Schaverien MV. Development of expertise in surgical training. J Surg Educ. (2010) 67:37–43. 10.1016/j.jsurg.2009.11.002 20421089

[48] Sinitsky DM Fernando B Berlingieri P. Establishing a curriculum for the acquisition of laparoscopic psychomotor skills in the virtual reality environment. Am J Surg. (2012) 204:367–76.e1. 10.1016/j.amjsurg.2011.11.010 22688107

[49] Palter VN Grantcharov TP. Development and validation of a comprehensive curriculum to teach an advanced minimally invasive procedure: a randomized controlled trial. Ann Surg. (2012) 256:25–32. 10.1097/SLA.0b013e318258f5aa 22664557

[50] Fransson BA Ragle CA. Assessment of laparoscopic skills before and after simulation training with a canine abdominal model. J Am Vet Med Assoc. (2010) 236:1079–84. 10.2460/javma.236.10.1079 20470069

[51] Levi O Michelotti K Schmidt P Lagman M Fahie M Griffon D. Comparison between training models to teach veterinary medical students basic laparoscopic surgery skills. J Vet Med Educ. (2016) 43:80–7. 10.3138/jvme.0715-109R 26752022

[52] Tapia-Araya AE Usón-Gargallo J Enciso S Pérez-Duarte FJ Díaz-Güemes Martin-Portugués I Fresno-Bermejo L. Assessment of laparoscopic skills in veterinarians using a canine laparoscopic simulator. J Vet Med Educ. (2016) 43:71–9. 10.3138/jvme.0315-034R1 26653288

[53] Cook DA Brydges R Ginsburg S Hatala R. A contemporary approach to validity arguments: a practical guide to Kane's framework. Med Educ. (2015) 49:560–75. 10.1111/medu.12678 25989405

[54] Durante Montiel I Lozano Sánchez JR Martínez González A Morales López S Sánchez M Evaluación de Competencias en Ciencias de la Salud. México: UNAM, Médica Panamericana (2012).

[55] Boza C Varas J Buckel E Achurra P Devaud N Lewis T. A cadaveric porcine model for assessment in laparoscopic bariatric surgery—a validation study. Obes Surg. (2013) 23:589–93. 10.1007/s11695-012-0807-9 23404238

[56] Cook DA Hatala R. Validation of educational assessments: a primer for simulation and beyond. Adv Simul Lond Engl. (2016) 1:31. 10.1186/s41077-016-0033-y 29450000 PMC5806296

[57] Noureldin YA Lee JY McDougall EM Sweet RM. Competency-Based Training and Simulation: Making a “Valid” Argument. J Endourol. (2018) 32:84–93. 10.1089/end.2017.0650 29437497

[58] Chen C-Y Elarbi M Ragle CA Fransson BA. Development and evaluation of a high-fidelity canine laparoscopic ovariectomy model for surgical simulation training and testing. J Am Vet Med Assoc. (2019) 254:113–23. 10.2460/javma.254.1.113 30668299

[59] Tapia-Araya AE Usón-Gargallo J Sánchez-Margallo JA Pérez-Duarte FJ Martin-Portugués ID-G Sánchez-Margallo FM. Muscle activity and hand motion in veterinarians performing laparoscopic training tasks with a box trainer. Am J Vet Res. (2016) 77:186–93. 10.2460/ajvr.77.2.186 27027713

[60] Sánchez-Hurtado MÁ Usón-Gargallo J Díaz-Güemes I Enciso S Sánchez-Peralta LF Sánchez-Fernández J Validación en simulación laparoscópica. Consideraciones metodológicas y de diseño. Arch Esp Urol. (2019) 79:904–14. 31697250

[61] Enciso Sanz S Evaluación de la Adquisición de Destrezas y Habilidades Quirúrugicas Durane La Formación en Cirugía Laparoscópica. Caceres: University of Extremadura (2013). Available online at: http://dehesa.unex.es/bitstream/handle/10662/800/TDUEX_2013_Enciso_Sanz.pdf?sequence=1&isAllowed=y

[62] Millán Núñez-Cortés J Palés Argullos JL Morán-Barrios J Principios de Educación Médica: Desde el Grado Hasta el Desarrollo Profesional. Madrid: Editorial Médica Panamericana (2015).

[63] García Murillo J Arias Correa ME *Diseño de Prototipo de Simulador Para Entrenamiento En Cirug*í*a Laparoscópica*. (2011) 5:13–19. Available online at: http://www.scielo.org.co/pdf/rinbi/v5n9/v5n9a03.pdf

[64] Barussaud M-L Roussel B Meurette G Sulpice L Meunier B Regenet N French intensive training course in laparoscopic surgery (HUGOFirst) on live porcine models: Validation of a performance assessment scale and residents' satisfaction in a prospective study. J Visc Surg. (2016) 153:15–9. 10.1016/j.jviscsurg.2015.10.005 26658147

[65] Macmillan AIM Cuschieri A. Assessment of innate ability and skills for endoscopic manipulations by the advanced dundee endoscopic psychomotor tester: predictive and concurrent validity. Am J Surg. (1999) 177:274–7. 10.1016/S0002-9610(99)00016-1 10219869

[66] Mason JD Ansell J Warren N Torkington J. Is motion analysis a valid tool for assessing laparoscopic skill? Surg Endosc. (2013) 27:1468–77. 10.1007/s00464-012-2631-7 23233011

[67] Smith CD Farrell TM McNatt SS Metreveli RE. Assessing laparoscopic manipulative skills. Am J Surg. (2001) 181:547–50. 10.1016/S0002-9610(01)00639-0 11513783

[68] Kondraske GV Hamilton EC Scott DJ Fischer CA Tesfay ST Taneja R. Surgeon workload and motion efficiency with robot and human laparoscopic camera control. Surg Endosc. (2002) 16:1523–7. 10.1007/s00464-001-8272-x 12098023

[69] Stylopoulos N Cotin S Maithel SK Ottensmeyer M Jackson PG Bardsley RS. Computer-enhanced laparoscopic training system (CELTS): bridging the gap. Surg Endosc. (2004) 18:8932. 10.1007/s00464-003-8932-0 15216861

[70] Datta V Chang A Mackay S Darzi A. The relationship between motion analysis and surgical technical assessments. Am J Surg. (2002) 184:70–3. 10.1016/S0002-9610(02)00891-7 12135725

[71] Lemos J Hernandez A Soto-Romero G. An instrumented glove to assess manual dexterity in simulation-based neurosurgical education. Sensors. (2017) 17:988. 10.3390/s17050988 28468268 PMC5469341

[72] Vassiliou MC Feldman LS Andrew CG Bergman S Leffondré K Stanbridge D. A global assessment tool for evaluation of intraoperative laparoscopic skills. Am J Surg. (2005) 190:107–13. 10.1016/j.amjsurg.2005.04.004 15972181

[73] Scott DJ Rege RV Bergen PC Guo WA Laycock R Tesfay ST. Measuring operative performance after laparoscopic skills training: edited videotape versus direct observation. J Laparoendosc Adv Surg Tech. (2000) 10:183–90. 10.1089/109264200421559 10997840

[74] Pérez-Duarte FJ Sánchez-Margallo FM Díaz-Güemes Martín-Portugués I Sánchez-Hurtado MÁ Lucas-Hernández M Usón Gargallo J. Ergonomía en cirugía laparoscópica y su importancia en la formación quirúrgica. Cir Esp. (2012) 90:284–1. 10.1016/j.ciresp.2011.04.021 21703603

[75] Maurin M Mullins RA Singh A Mayhew PD. A systematic review of complications related to laparoscopic and laparoscopic-assisted procedures in dogs. Vet Surg. (2020). 10.1111/vsu.13419. [Epub ahead of print]. 32333685

